# Geometric decoupling of epidermal melanin and dermal hemoglobin signals using coaxial and spatially resolved NIRS

**DOI:** 10.1117/1.JBO.31.10.107001

**Published:** 2026-10-08

**Authors:** Vedanshi Patel, Devang Vyas, John Hanks, Amir Tofighi Zavareh

**Affiliations:** a Texas A&M University, Department of Biomedical Engineering, College Station, Texas, United States; b Texas A&M University, Center for Remote Health Technologies and Systems, College Station, Texas, United States; c Texas A&M University, Department of Engineering Technology & Industrial Distribution, College Station, Texas, United States

**Keywords:** near-infrared spectroscopy, tissue oxygen saturation, peripheral arterial disease, melanin, spatially resolved spectroscopy, Monte Carlo simulation, machine learning, XGBoost, mutual information

## Abstract

**Significance:**

Epidermal melanin can confound NIRS measurements of tissue oxygen saturation and reduce accuracy across skin tones.

**Aim:**

We evaluated whether a coaxial detector can provide a superficial reference that improves absolute ${\mathrm{StO}}_{2}$ estimation when combined with spatially resolved measurements.

**Approach:**

Monte Carlo simulations of multilayer skin were evaluated using mutual information and XGBoost regression, with entire melanin levels held out during validation. Three detector configurations were compared: coaxial only, surface only, and the combined geometry. A secondary experimental analysis examined absolute and baseline referenced oxygenation prediction using validation that excluded one recording at a time.

**Results:**

The combined coaxial plus surface detector configuration reduced average ${\mathrm{StO}}_{2}$ RMSE from 11.34 to 5.41 percentage points, corresponding to a 52.3% relative reduction based on the displayed fold results. Mutual information analysis showed that the coaxial detector carried substantial information about melanin while carrying negligible direct information about ${\mathrm{StO}}_{2}$. In the experimental analysis, baseline-referenced features improved pooled agreement, but the recording-based validation did not establish generalization to entirely unseen participants.

**Conclusions:**

These findings support future experimental validation of integrated coaxial plus spatially resolved probes for melanin-robust absolute tissue oxygenation monitoring.

## 1 Introduction

Tissue oxygen saturation (${\mathrm{StO}}_{2}$) is a noninvasive spectroscopic indicator of local microvascular oxygen delivery and demand, with relevance to ischemia, wound healing, flap monitoring, and peripheral vascular assessment. Near-infrared spectroscopy (NIRS) is widely used for ${\mathrm{StO}}_{2}$ estimation because oxyhemoglobin and deoxyhemoglobin exhibit distinct absorption spectra in the red to near-infrared range.^1^^–^^9^

Continuous wave NIRS (CW-NIRS) typically estimates ${\mathrm{StO}}_{2}$ from wavelength-dependent attenuation using modified Beer-Lambert relationships. However, absorption and scattering cannot be independently recovered from intensity alone, and most compact systems rely on assumed or fixed scattering behavior. Because epidermal melanin introduces broadband superficial absorption and scattering, intersubject pigmentation variation can be misattributed to hemoglobin oxygenation, degrading absolute ${\mathrm{StO}}_{2}$ accuracy across skin tones.^10^^–^^19^

Prior approaches to pigmentation correction include empirical skin tone correction, time-domain and frequency-domain NIRS (TD-NIRS, FD-NIRS), spatial frequency domain imaging (SFDI), multilayer inverse modeling, and machine learning-based spectral correction. These methods can improve robustness but may require larger instrumentation, additional assumptions, or correction models that remain dependent on the measured spectra. These limitations motivate a probe-level strategy that physically separates superficial pigmentation information from deeper hemoglobin-sensitive sampling within a topology that can be implemented in a portable point-of-care design.

Spatially resolved spectroscopy (SRS) partially addresses this problem by using the spatial decay of diffuse reflectance across source-detector separations to estimate absorption and reduced scattering. However, laterally displaced channels still collect photons that traverse the epidermis, so melanin remains present in every detector signal. Thus, SRS reduces but does not eliminate ambiguity between superficial pigmentation and deeper hemoglobin absorption.^20^^–^^25^

Coaxial detector configurations have been previously proposed as a means of isolating superficial optical contributions and detecting melanin levels accurately.^26^ However, a coaxial channel alone is insufficient for recovering separated oxygenation information. The intensity collected at zero lateral offset is dominated by superficial backscattering and epidermal absorption, as the associated photon path length is too short to capture meaningful hemoglobin-dependent attenuation from the dermal vasculature.^21^^,^^27^ Without a complementary distal measurement, the coaxial channel cannot independently decompose epidermal and vascular contributions without reintroducing the same model-based assumptions that limit existing correction strategies.^11^^,^^24^ This limitation is also relevant to a broader class of standoff diffuse reflectance and hyperspectral imaging systems that estimate tissue oxygenation from spectral hypercubes. Although these systems provide rich spatial and spectral information, many rely primarily on remitted superficial reflectance without an explicit depth-separated detector geometry.

This limitation is not only theoretical. Our group’s prior measurements utilized a compact coaxial LED photodetector device to track melanin levels and categorize baseline relative oxygenation dynamics during vascular occlusion testing.^27^^–^^30^ The present study is distinct from our prior work, which evaluated a handheld coaxial multispectral device (PhasorDetect) for baseline-relative monitoring during vascular occlusion testing. Here, we use that limitation as motivation for a new hybrid geometry. We hypothesize that a coaxial channel provides a superficial melanin-sensitive reference, while spatially resolved detectors provide deeper hemoglobin-sensitive measurements for absolute ${\mathrm{StO}}_{2}$ estimation.^31^^–^^33^

This hypothesis is tested through a systematic *in silico* study using three-dimensional Monte Carlo simulations of a multilayer skin model with embedded dermal capillaries,^34^^,^^35^ with gradient-boosted regression models (XGBoost)^36^ trained and validated using a leave-one-group-out (LOGO) cross-validation scheme grouped by melanin level *in silico*. This study does not rely only on the results of a specific ML algorithm to infer broader physiological intuition. Instead, the study uses model-agnostic parameters such as mutual information (MI) to understand the underlying mechanisms of light-tissue interactions in these different geometries.

This study examines the optical and informational rationale for adding a coaxial superficial reference channel to SRS and includes an exploratory *in vivo* comparison of absolute and baseline-referenced prediction from coaxial reflectance under recording-based validation.

## 2 Methods

### 2.1 Monte Carlo Tissue Model

A three-dimensional (3D) computational model of the human volar forearm was developed using MCMATLAB to simulate photon transport through a multilayer skin geometry.^37^ The model domain measured 2.5×2.5×2.0  cm and comprised four anatomically distinct layers: epidermis, dermis, dermal vasculature, and subcutaneous fat. The domain was discretized into 100 bins along each of the x- and y-axes and 1000 bins along the z-axis. The tissue block geometry is shown in Fig. 1, and the optical properties of each layer are shown in Fig. 2.

**Fig. 1:**
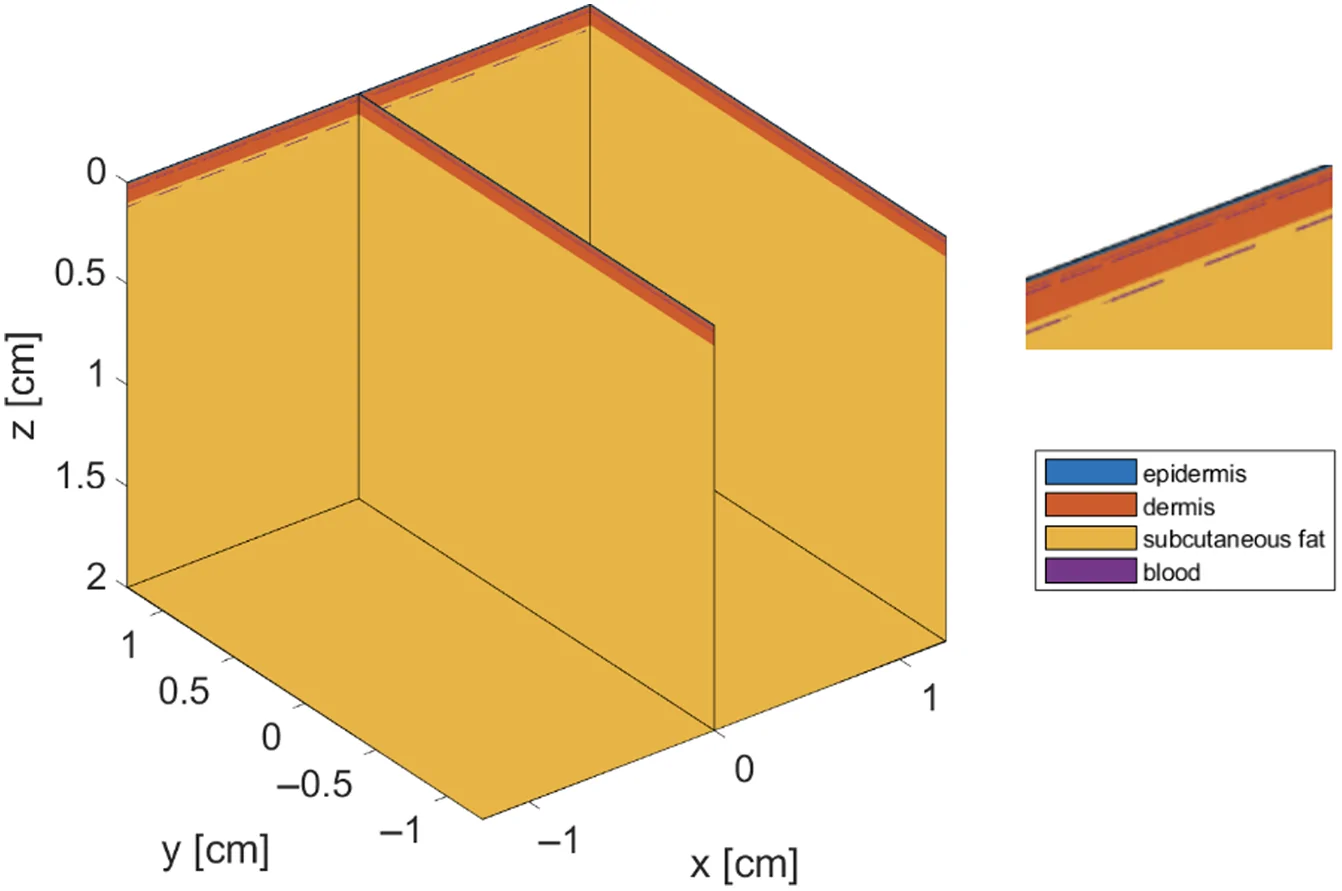
Geometry of the human forearm skin model (2.5×2.5×2.0  cm) consisting of four tissue layers, each represented by a different color.

**Fig. 2:**
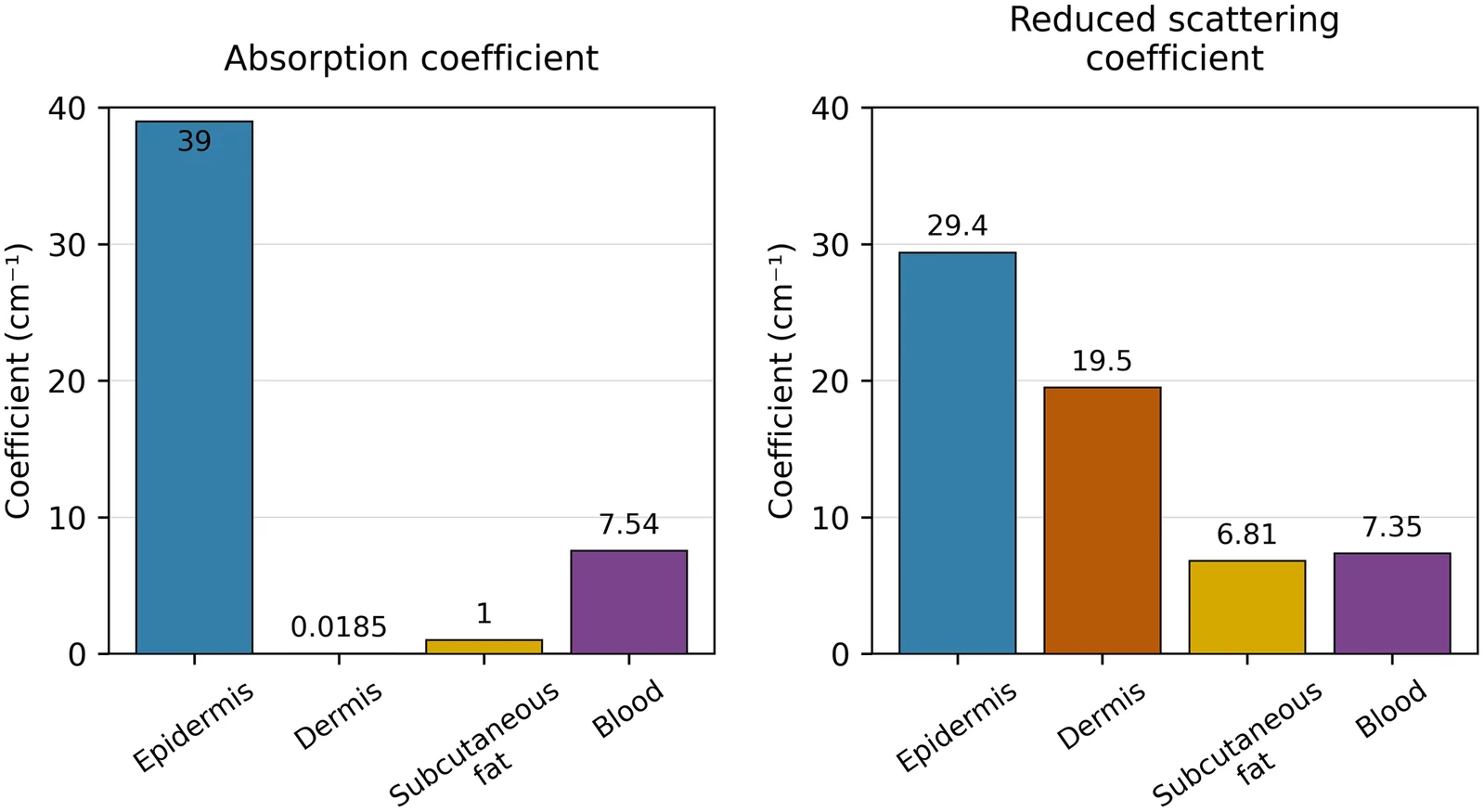
Absorption and reduced scattering coefficients at 680 nm for the four tissue layers, representing 46.95% ${\mathrm{StO}}_{2}$, 16% melanin volume fraction, and 73  μm epidermal thickness. Both panels use an ordinate range of 0 to $40\;{\mathrm{cm}}^{-1}$; horizontal guides support comparison.

To estimate the optical properties of biological tissue, MCMATLAB combines standard constituent terms following Jacques.^38^ The total absorption coefficient $\mu_{a}(\lambda )$, in ${\mathrm{cm}}^{-1}$, is calculated using Eq. (1):

$$\mu_{a}(\lambda )=[B]\cdot ([S]\cdot \mu_{a},{\mathrm{HbO}}_{2}(\lambda )+(1-[S])\cdot \mu_{a},\mathrm{Hb}(\lambda ))\;+[W]\cdot \mu_{a},\text{water}(\lambda )+[F]\cdot \mu_{a},\text{fat}(\lambda )+[M]\cdot \mu_{a},\text{melanin}(\lambda ).$$ (1)

Here [B], [W], [F], and [M] denote the dimensionless volume fractions of blood, water, fat, and melanin, respectively. [S] denotes oxygen saturation as a dimensionless fraction. All fractions in Eq. (1) range from 0 to 1; for example, 30% is entered as 0.30. Brackets identify fractional quantities and do not indicate percent units. The symbol λ denotes wavelength, and $\mu_{a}$, i(λ) is the absorption coefficient of constituent i.

The reduced scattering coefficient $\mu_{s}^{\prime }(\lambda )$, in ${\mathrm{cm}}^{-1}$, is calculated using Eq. (2):

$$\mu_{s}^{\prime }(\lambda )=a^{\prime }[f_{\text{Ray}}\cdot (\lambda /500\;\mathrm{nm}{)}^{-4}+(1-f_{\text{Ray}})\cdot (\lambda /500\;\mathrm{nm}{)}^{-b_{\text{Mie}}}].$$ (2)

Here, $a^{\prime }$ is the reduced scattering coefficient at 500 nm, fRay is the Rayleigh scattering fraction, and bMie is the Mie scattering power. The wavelength ratio is dimensionless.

#### 2.1.1 Epidermis

Epidermal absorption was modeled as a function of wavelength, melanin volume fraction, water content, and negligible fat content. Blood content was set to zero. Reduced scattering was calculated following Jacques with a reference value of $40\;{\mathrm{cm}}^{-1}$ at 500 nm, a Rayleigh fraction of zero, a Mie scattering power of 1.0, and an anisotropy factor g=0.9. ^37^^,^^39^

#### 2.1.2 Dermis and dermal vasculature

Dermal blood volume fraction was set to 0.002, and ${\mathrm{StO}}_{2}$ was varied as the target variable. Dermal water content was 0.65, with melanin and fat set to zero. Reduced scattering used a $42.4\;{\mathrm{cm}}^{-1}$ reference at 500 nm, a Rayleigh fraction of 0.62, and g=0.9. Vascular absorption was computed using pure blood with 95% water content, and vascular reduced scattering was set to $10\;{\mathrm{cm}}^{-1}$.

#### 2.1.3 Subcutaneous fat

Subcutaneous fat optical properties were assigned from published wavelength-dependent absorption and reduced scattering values and served as the lower tissue boundary.^40^

### 2.2 Simulation Parameter Space

Four illumination wavelengths were simulated: 680, 720, 760, and 800 nm. Epidermal melanin volume fraction varied across nine levels from 5% to 45% in increments of 5%, encompassing lightly to deeply pigmented skin.^39^ Epidermal thickness was set to three values: 65, 80, and 95  μm, consistent with the range reported for the volar forearm.^41^ ${\mathrm{StO}}_{2}$ varied from 20% to 80% across 20 evenly spaced intervals. A uniform refractive index of 1.4 and an anisotropy factor of 0.9 were assumed for all tissue layers to exclude differences in reflection and refraction at internal tissue interfaces and isolate the effects of the investigated optical and geometric parameters. Each unique parameter combination was simulated five times to reduce the influence of Monte Carlo noise.

### 2.3 Probe Configurations

Two complementary probe configurations, a coaxial and a spatially resolved detector design, were evaluated in this study across a systematic multidimensional parameter sweep. Figure 3 provides an overview of the physical arrangement of the cone assembly, illumination source, and detector boards used in both configurations. Figure 4 shows the implementation of the two probe configurations in the MCMATLAB simulation. Detailed descriptions of these configurations are discussed in Secs. 2.3.1 and 2.3.2.

**Fig. 3:**
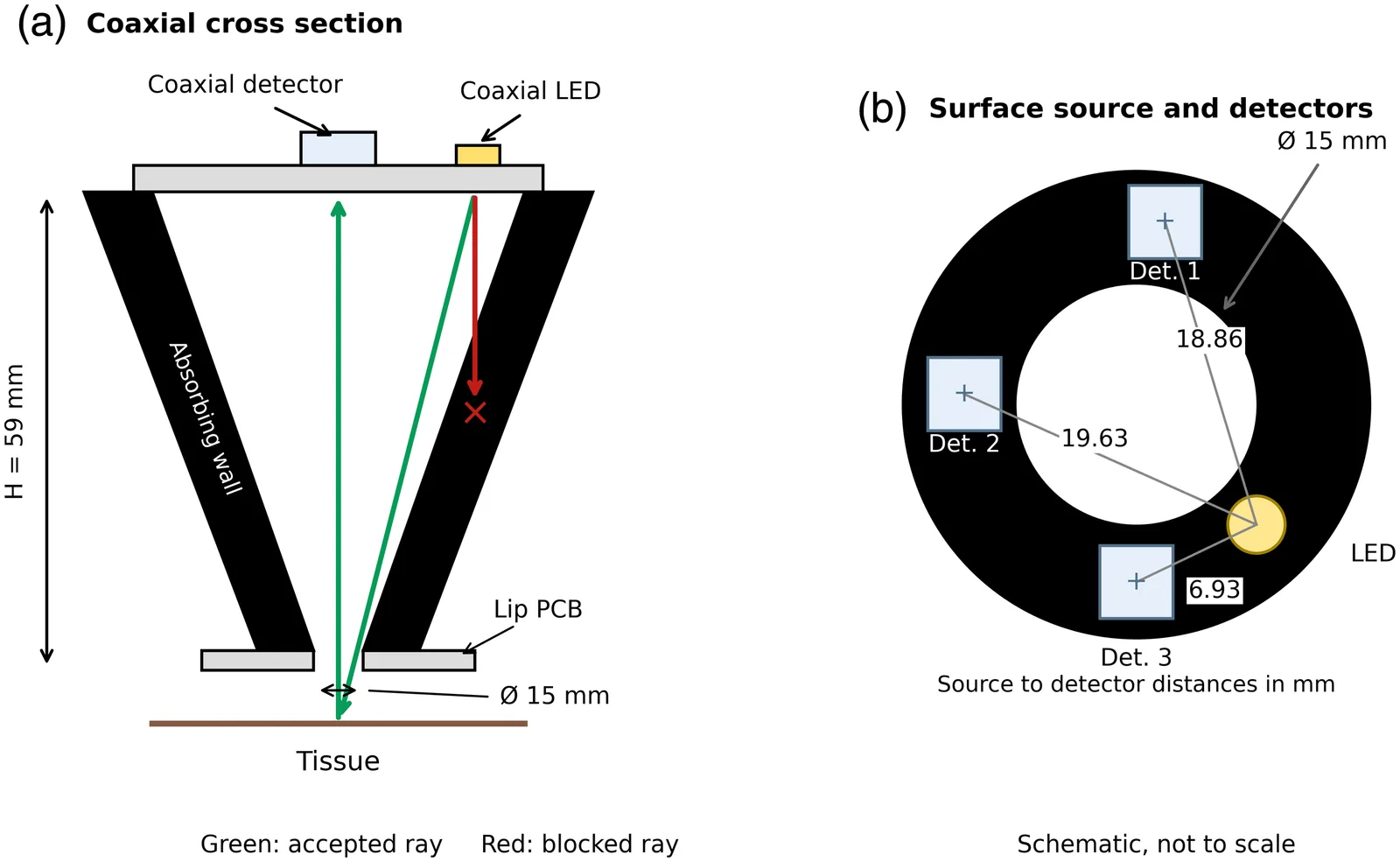
Vector schematic of the complementary probe geometries, not to scale. (a) Coaxial cross-section showing absorbing cone walls, the 59 mm height, the 15 mm aperture, and the optical components. Green arrows indicate accepted rays, and the red arrow indicates a ray blocked by the cone wall. (b) Surface detector and LED positions on the absorbing annulus. Source-to-detector distances are shown in millimeters. The schematic retains the dimensions and component relationships of the original design while omitting incidental CAD assembly detail.

**Fig. 4:**
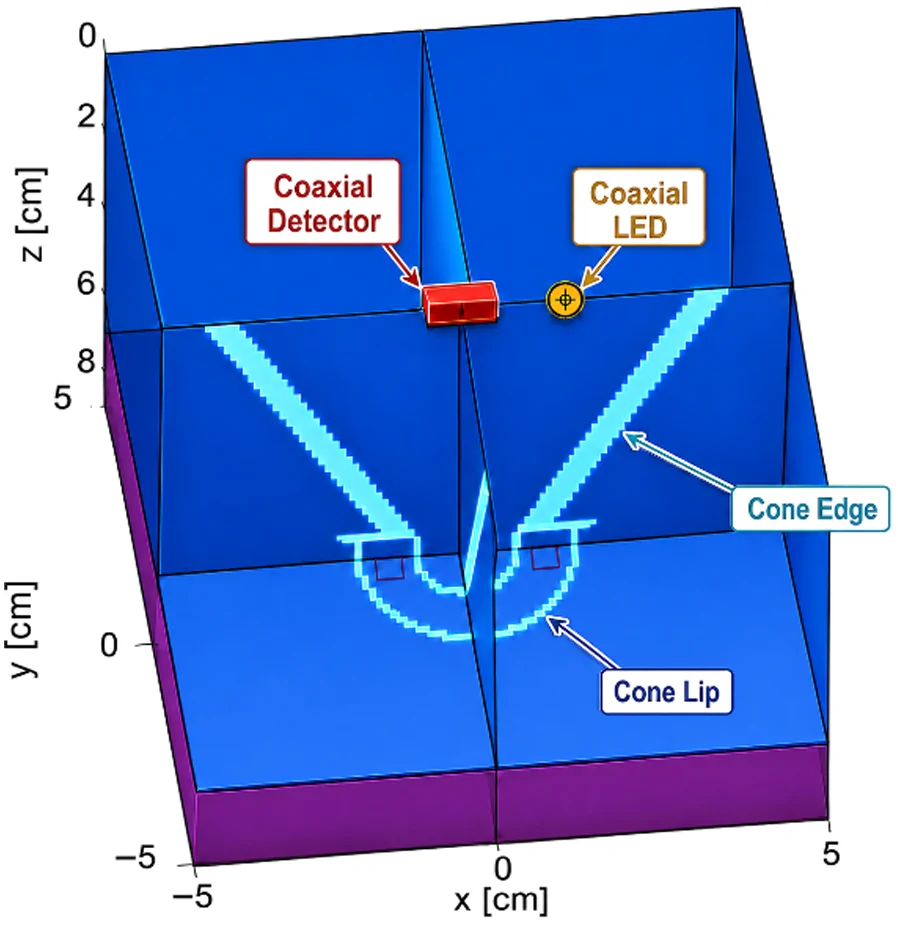
Placement of the coaxial and SRS configurations in the MCMATLAB simulation. The location of the coaxial detector is indicated by the red rectangle, whereas the coaxial LED source is represented by the yellow circle. The absorbing cone boundary is highlighted in blue, and the underlying tissue volume is shown in purple. The SRS configuration is simulated at the lip of the cone, highlighted by two boxes.

#### 2.3.1 Coaxial illumination detector configuration

The coaxial detector architecture is designed to preferentially collect photons backscattered from the superficial tissue layers while suppressing specular reflection. This design uses a centrally co-aligned source and detector configuration positioned inside an absorbing cone, as shown in Fig. 3(a). The coaxial LED and the detector are at a height of 59 mm and have an opening aperture of 15 mm diameter. The specific height and aperture were selected such that the emission angles of the LED produced an illumination footprint concentrated near the perimeter of the aperture at the tissue interface. In the hardware, the inside wall of the cone is coated with Black 2.0 paint to reduce the amount of light reflected directly on the detector. The absorbing cone (Fig. 3) was imported into the MCMATLAB as a voxel grid (Fig. 5) and assigned high absorption ($100\;{\mathrm{cm}}^{-1}$) to suppress specularly reflected photons.

**Fig. 5:**
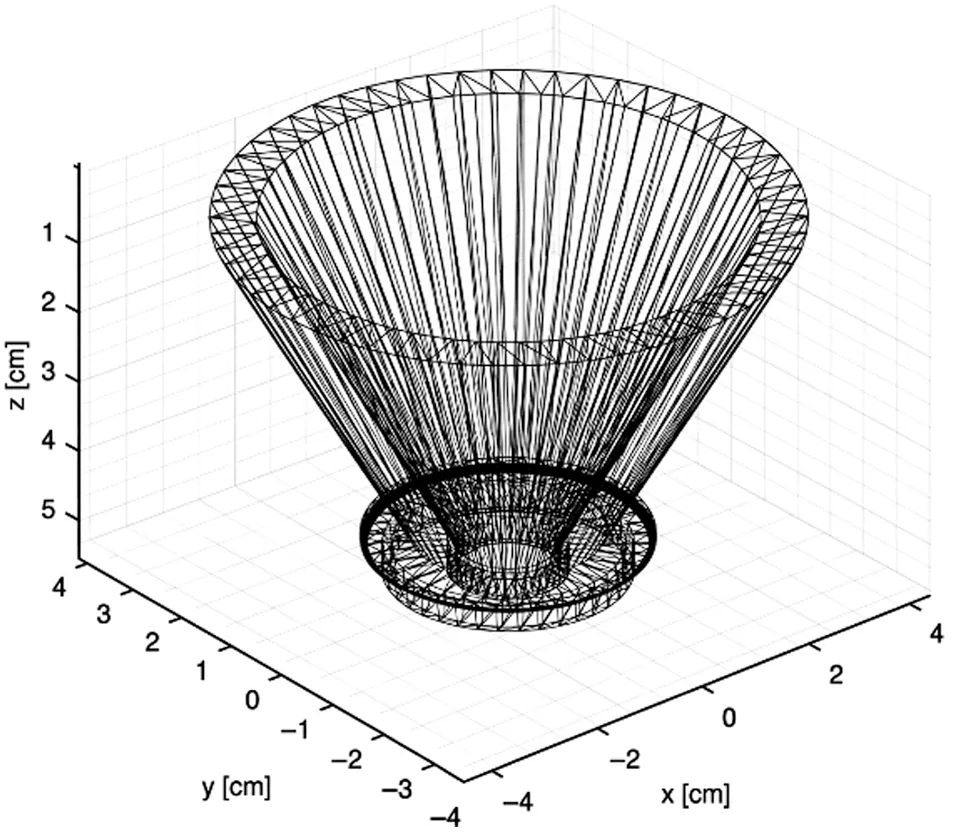
Three-dimensional voxelized representation of the absorbing cone was imported into the MCMATLAB simulation program. The cone walls are assigned high absorption coefficients on both the inner and outer surfaces to suppress specular reflections and direct optical leakage, allowing the diffused, scattered photons from the tissue through the aperture to reach the detector.

The source was modeled as a circular LED with a 1.2 mm radius and a custom angular emission profile with a 20-deg half-angle (Fig. 6). Each simulation launched $1.5\times 10^{8}$ photon packets.

**Fig. 6:**
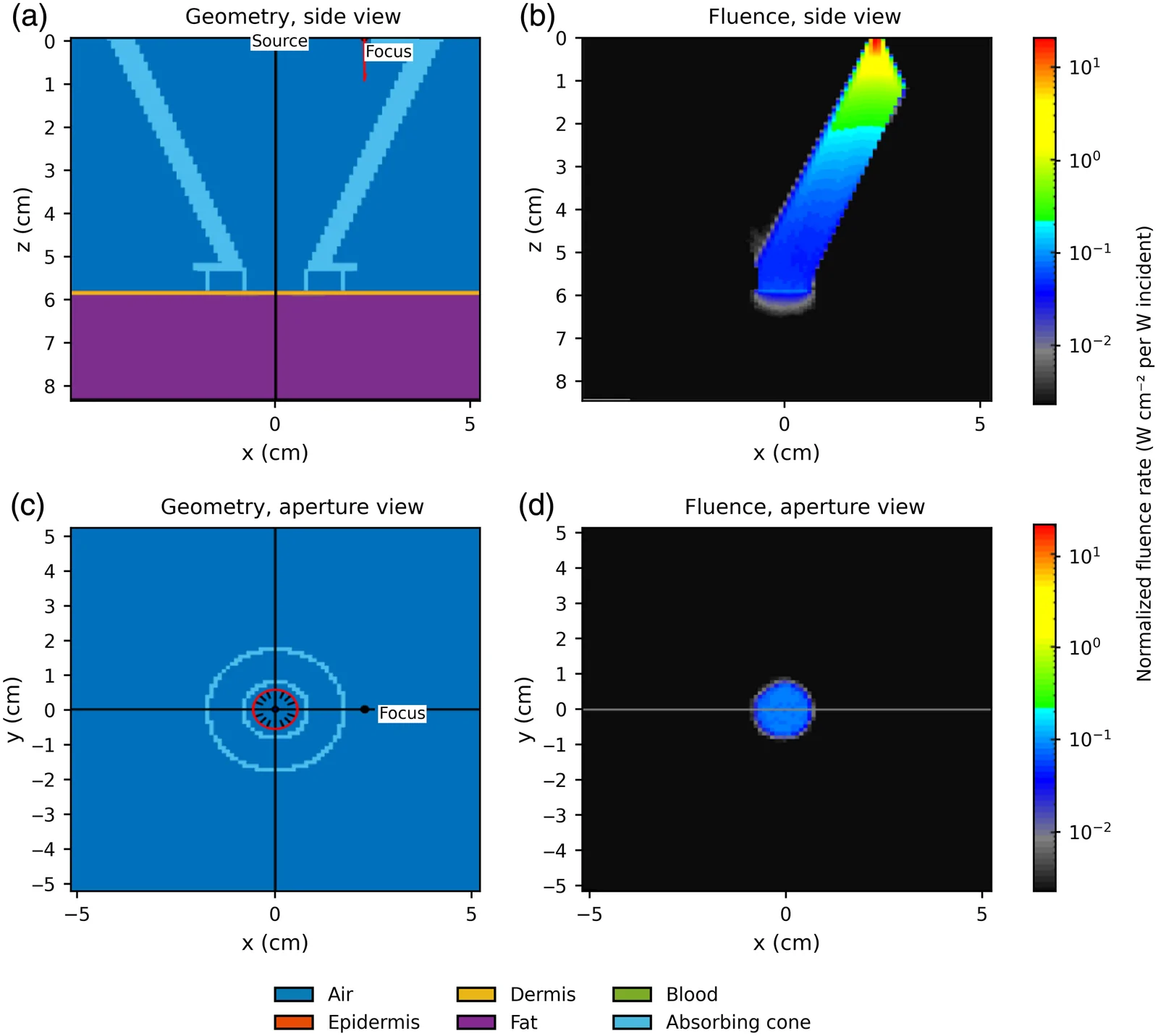
Simulated illumination distribution at the cone tissue interface. Panels (a) and (c) show the modeled geometry in side and aperture views, respectively. Panels (b) and (d) show the corresponding normalized fluence rate on logarithmic color scales. Source and focus annotations are retained in the geometry views. The simulation image content and color scales are preserved; titles, axes, and the shared material legend have been reformatted for readability.

The coaxial detector was positioned at the center of the cone aperture, at surface coordinates (0, 0), capturing photons enriched for short path lengths and high-angle backscattered trajectories within the superficial tissue volume.^42^

#### 2.3.2 Spatially resolved spectroscopy configuration

For the spatially resolved spectroscopy (SRS) configuration, the cone lip circumference was divided into nine feasible detector regions (Fig. 7), each representing an ∼4×5  mm detector footprint with a single light source fixed in position. All 84 possible three-detector combinations were evaluated using the same simulated biological parameter space as the rest of this study. For each triplet, raw detector fluence values were used to train an XGBoost regressor for ${\mathrm{StO}}_{2}$ prediction, and leave-one-melanin-level-out cross-validation MSE was used as the optimization criterion. The triplet with the lowest cross-validated MSE was selected as the final surface detector configuration. This ensured that the SRS baseline reflected an optimized surface-detector arrangement rather than a disadvantaged comparator.

**Fig. 7:**
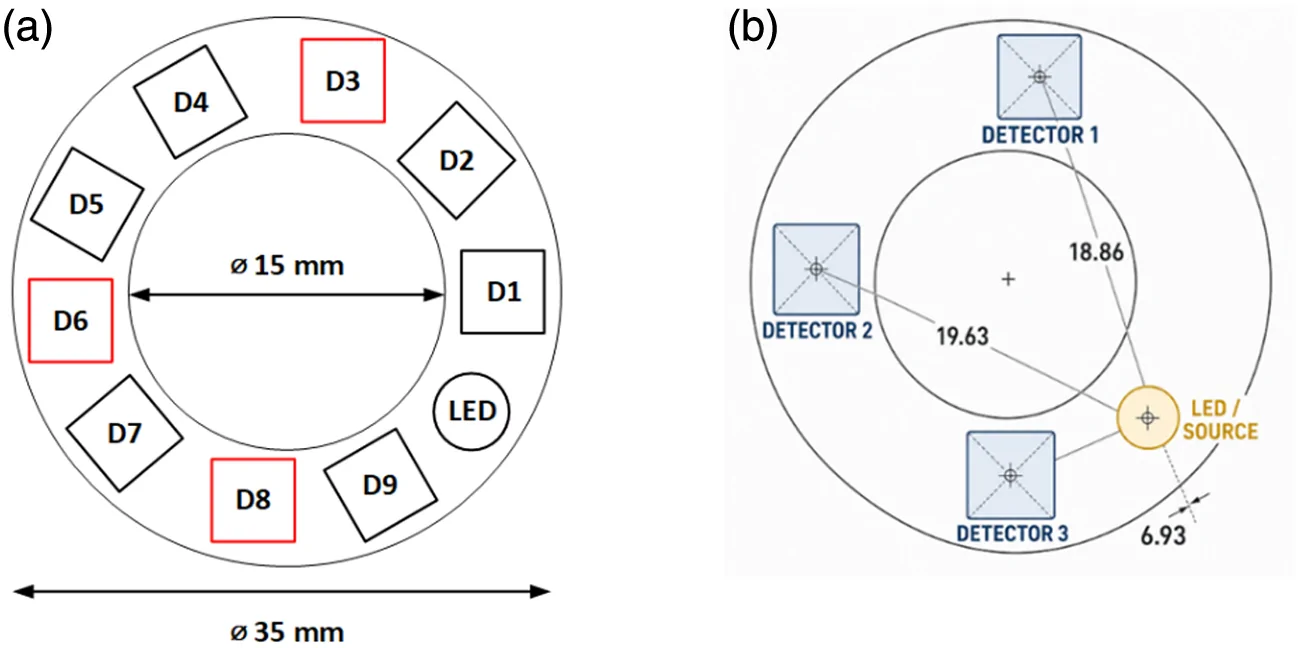
Visual representation of the nine detector regions and the selected regions for the simulations programmed in MCMATLAB for the SRS configuration. (A) Nine feasible detector regions (D1-D9) distributed around the 15 mm cone aperture. Selected detector positions are highlighted in red. (B) The three selected detector locations and their distances from the fixed LED source.

For each probe configuration, the full sweep across wavelength, melanin fraction, epidermal thickness, ${\mathrm{StO}}_{2}$, and replicate was simulated (Table 1). Detector fluence values were extracted from the three-dimensional fluence arrays and merged into a master feature database indexed by biological and optical parameters. This database was used for mutual information analysis, feature engineering, model training, and cross-validation.

**Table 1:** Summary of the optimization parameters used to select the combination of three surface spatially resolved detectors.

Category	Parameter	Value
Candidate detector regions	Number of feasible regions	9
Detector footprint	Region size	∼4×5 mm
Selected detectors	Number of detectors per configuration	3
Search strategy	Detector combinations evaluated	84
Biological parameters	Melanin fractionepidermal thickness ${\mathrm{StO}}_{2}$	5% to 45%65, 80, and 95 μm 20% to 80%
Spectral parameters	Wavelengths	680, 720, 760, 800 nm
Optimization output	Spatially resolved detector distances	6.93, 18.86, 19.63 mm

#### 2.3.3 Rationale for complementary probe geometries

The coaxial and spatially resolved configurations were designed to provide complementary sensitivity to superficial and deeper tissue layers. Prior literature states that an increase in the source-to-detector distance can increase the sampling depth.^43^ Figure 8 conceptually illustrates the expected photon trajectories associated with each geometry. In the coaxial configuration, the absorbing cone’s 15 mm aperture limits the accepted photon trajectories. Although the coaxial source and detector are aligned about the same central axis, illumination and collection occur over the finite 15 mm aperture. The coaxial signal, therefore, represents an average over a distribution of short photon paths with different superficial sampling depths, rather than a single zero-separation trajectory. In comparison, the SRS configuration uses three defined source–detector separations, providing more distinct depth-dependent sampling volumes.

**Fig. 8:**
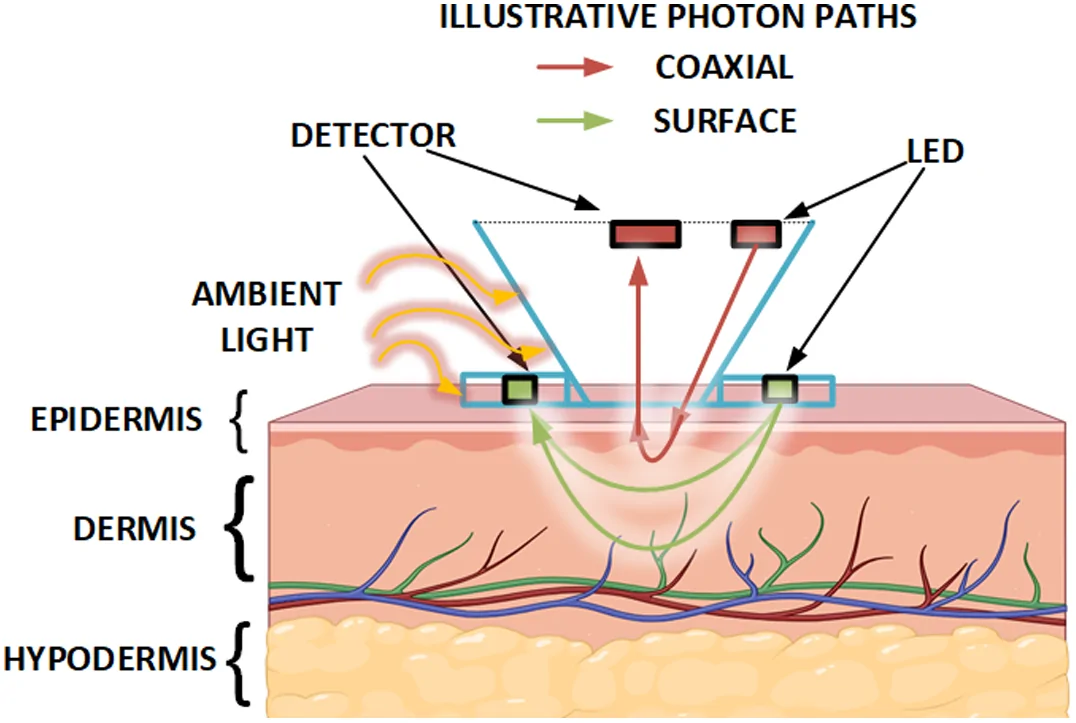
Conceptual illustration of the photon trajectories associated with the coaxial and spatially resolved probe geometries. The coaxial configuration (red) has photons that emerge from the cone’s circular aperture and are collected back through the same aperture. The SRS configuration (green) samples photons that travel deeper through the tissue. The cone walls (blue) reject ambient light and help increase the sensitivity to light collected from the skin.

### 2.4 Mutual Information Analysis

Mutual information analysis was used to quantify the dependence between detector signals and each target variable before model training. Because MI captures nonlinear statistical dependence and is independent of model architecture, it provides a model-agnostic measure of information content. Joint MI was estimated using the Kraskov-Stögbauer-Grassberger nearest neighbor estimator with k=5 after feature standardization. MI was computed for three detector groupings: surface detectors only, coaxial detectors only, and surface plus coaxial detectors.^44^

To isolate the contribution of the coaxial channel to ${\mathrm{StO}}_{2}$ beyond the surface array, the conditional mutual information was calculated using Eq. (3):

$$I(X_{C};{\mathrm{StO}}_{2}|X_{S})=I((X_{S},X_{C});{\mathrm{StO}}_{2})-I(X_{S};{\mathrm{StO}}_{2}).$$ (3)

Here, I denotes mutual information, X denotes the coaxial detector features, and $X_{s}$ denotes the surface detector features. The corresponding conditional information about melanin was calculated using Eq. (4):

$$I(X_{C};M|X_{S})=I((X_{S},X_{C});M)\;-I(X_{S};M).$$ (4)

### 2.5 Feature Engineering

Raw detector fluence values are not suitable as direct inputs for ${\mathrm{StO}}_{2}$ prediction. The photon flux is a composite signal shaped exponentially by wavelength-dependent scattering, optical path length elongation, and broadband absorption by static chromophores such as melanin. A physics-informed feature engineering pipeline was developed.

#### 2.5.1 Optical density conversion

Raw fluence values were converted to optical density (OD) using Eq. (5):

$${\mathrm{OD}}_{\lambda }=-\ln(I_{\lambda }),$$ (5)

where ${\mathrm{OD}}_{\lambda }$ is the optical density at wavelength λ, and $I_{\lambda }$ is the normalized detector fluence at wavelength λ, and is treated as dimensionless because it is normalized by the simulation source intensity. In the logarithmic domain, layered absorption contributions can be treated approximately additively under the modified Beer-Lambert framework. This transformation was used before baseline subtraction, spectral derivative calculation, and spatial normalization.^45^

#### 2.5.2 Spectral gradient

A spectral difference ΔODλ, 800 was computed between each selected wavelength and the 800 nm reference using Eq. (6):

$$\Delta {\mathrm{OD}}_{\lambda ,800}={\mathrm{OD}}_{800}-{\mathrm{OD}}_{\lambda }.$$ (6)

Between 680 and 760 nm, the molar extinction coefficient of HHb is substantially elevated relative to ${\mathrm{HbO}}_{2}$, whereas at 800 nm the two species converge at the isosbestic point. The difference, therefore, functions as a first-order oxygenation indicator independent of static attenuation offsets from tissue pigmentation.

#### 2.5.3 Coaxial spatial normalization

Surface detector optical densities were referenced to the coaxial optical density at the same wavelength using Eq. (7), to reduce shared superficial attenuation, including absorption associated with epidermal melanin:

$${\mathrm{OD}}_{D,\lambda ,\text{normcoaxial}}={\mathrm{OD}}_{\mathrm{SRS},\lambda }-{\mathrm{OD}}_{\text{coaxial},\lambda }.$$ (7)

#### 2.5.4 Isosbestic normalization

Signals at each wavelength were normalized by the 800 nm channel using Eq. (8). The small numerical stabilizer $\varepsilon =1\times 10^{-25}$ was included where specified to avoid division by zero.

$$I_{\lambda ,\text{norm}800}=\frac{I_{\lambda }}{I_{800}+\varepsilon }.$$ (8)

#### 2.5.5 Dual normalization

The dual normalization feature was calculated using Eq. (9):

$$I_{\lambda ,\text{dual}}=\frac{I_{\mathrm{SRS},\lambda }}{I_{\text{coaxial},\lambda }\cdot I_{\mathrm{SRS},800}+\varepsilon }.$$ (9)

These features were designed to reduce sensitivity to both pigmentation-related attenuation and regional blood volume variation.

#### 2.5.6 Wide gap second derivative spectroscopy

Wide gap second difference features were computed across the 680 to 800 nm range using Eqs. (10) and (11), to suppress constant offsets and slowly varying backgrounds while retaining spectral curvature associated with hemoglobin.^46^ The common squared wavelength spacing is omitted because the two terms are subsequently used as a ratio.

$$d_{720}^{2}={\mathrm{OD}}_{760}-2\cdot {\mathrm{OD}}_{720}+{\mathrm{OD}}_{680},$$ (10)

$$d_{760}^{2}={\mathrm{OD}}_{800}-2\cdot {\mathrm{OD}}_{760}+{\mathrm{OD}}_{720}.$$ (11)

#### 2.5.7 Scaling by the second derivative ratio

The ratio in Eq. (12) was used as a dimensionless curvature descriptor to reduce dependence on common optical path length and hemoglobin scaling factors:

$$D_{720,\text{scaled}}^{2}=\frac{d_{720}^{2}}{d_{760}^{2}+\varepsilon }.$$ (12)

### 2.6 Machine Learning Model, Hyperparameter Optimization, and Cross-Validation

Gradient-boosted regression trees were implemented using XGBoost with a squared-error minimization objective. This model class was selected to capture nonlinear relationships between engineered optical features and ${\mathrm{StO}}_{2}$ while allowing regularization through tree depth, subsampling, and L1 and L2 penalties. Hyperparameter selection used a randomized search over eight hyperparameters: number of estimators [500, 1000, 1500], learning rate [0.01, 0.05, 0.10], maximum tree depth [3, 5, 7, 9], row subsampling fraction [0.70, 0.80, 0.90], column subsampling fraction per tree [0.70, 0.80, 0.90], L1 regularization [0, 0.1, 0.5, 1.0], L2 regularization [1.0, 5.0, 10.0], and minimum split loss gamma [0, 0.1, 0.5]. Sixty configurations were sampled per search instance, parallelized across nine independent array jobs with distinct random seeds, yielding 540 unique configurations evaluated in total. Each configuration was scored using negative mean squared error averaged across cross-validation folds. Leave-one-group-out (LOGO) cross-validation was performed using melanin volume fraction as the grouping variable. In each fold, all samples from one melanin level were held out for testing, and the model was trained on all remaining melanin levels. This design avoids leakage across pigmentation groups and tests generalization to unseen melanin conditions.

### 2.7 Experimental Coaxial Reflectance Analysis

To connect the simulation results with measured data, we performed an exploratory secondary analysis of reflectance collected with PhasorDetect, an eight wavelength handheld multispectral device operating from 525 to 940 nm with a coaxial LED and photodetector geometry. The data were collected during vascular occlusion testing with synchronized Hutchinson InSpectra ${\mathrm{StO}}_{2}$ values, following the previously described protocol 27. The present analysis compares absolute ${\mathrm{StO}}_{2}$ prediction with baseline referenced $\Delta {\mathrm{StO}}_{2}$ prediction using engineered coaxial reflectance features. It examines the limitations of coaxial measurements as motivation for the hybrid geometry, without directly validating the simulated surface detector array.

Fourteen subjects consented and underwent vascular occlusion testing with synchronized PhasorDetect and Hutchinson InSpectra recordings. A preocclusion baseline was collected, followed by cuff-induced ischemia and reperfusion. Because the two probes were not co-located, the comparison was interpreted as trend and baseline relative agreement rather than strict point-wise equivalence.

The experiment was approved by the Texas A&M University Institutional Review Board (IRB2023-0596). The experimental setup is shown in Fig. 9.

**Fig. 9:**
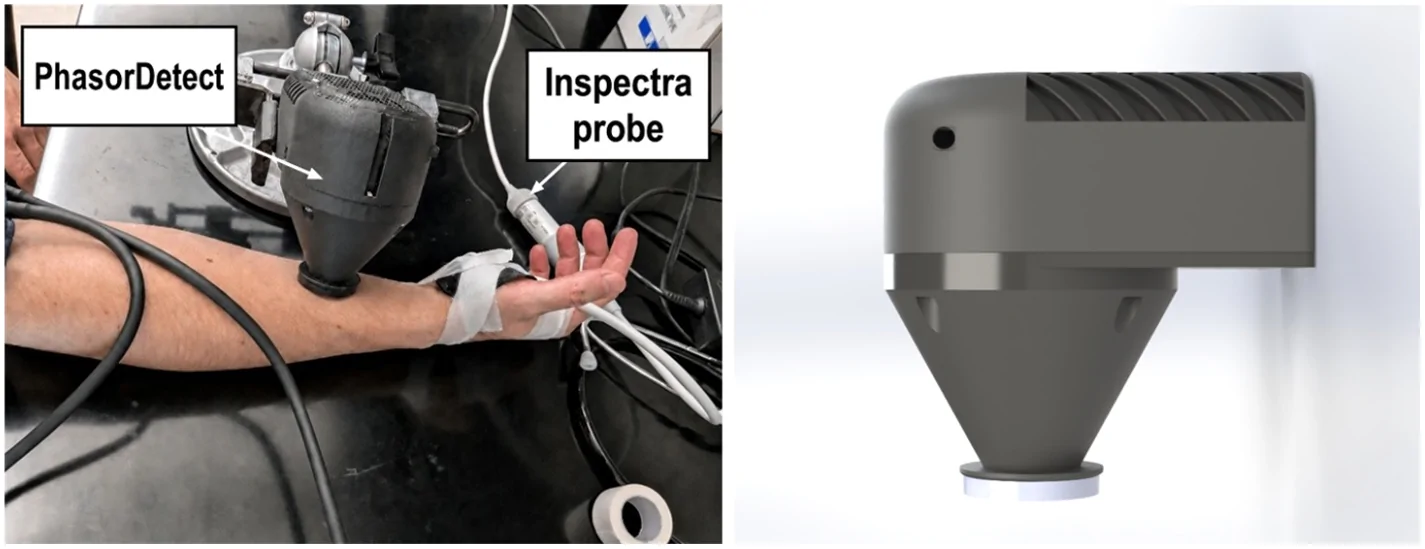
Left: placement of the PhasorDetect device and the Hutchinson InSpectra probe during the vascular occlusion test (VOT). Right: 3D rendering of the PhasorDetect device.

The experimental dataset comprised 28 recordings from 14 participants, with separate left and right recordings. For each recording, spreadsheet rows 10 through 300 were retained, giving 291 observations per recording and 8148 observations in total. The first 100 retained observations defined each recording’s baseline. The absolute model used 11 features derived from reflectance at 630, 680, 780, and 810 nm: optical densities, a spectral gradient, three ratios normalized to 810 nm, two second derivatives, and their ratio. Experimental optical density was calculated using the negative base 10 logarithm of reflectance, whereas Eq. (5) defines the natural logarithm convention for the simulation features. The second derivatives were obtained from quadratic fits to the unequal wavelength triplets. The shifted model used changes in optical density and reflectance relative to the recording baseline, with $\Delta {\mathrm{StO}}_{2}$ defined relative to the corresponding reference baseline.

Experimental regression used MATLAB LSBoost with 1000 learning cycles, a learning rate of 0.05, at most seven splits per tree, and a minimum leaf size of five. The script requested row and predictor sampling, with compatibility fallbacks. Each fold excluded all observations from one recording, identified by participant and side. Thus, the participant’s other recording remained eligible for training. This differs from the simulated XGBoost analysis and is not validation on entirely unseen participants. RMSE, MAE, and $R^{2}$ were pooled across the excluded recordings. The saved figure coordinates were used to verify the reported experimental metrics without refitting the models.

## 3 Results

### 3.1 Mutual Information Analysis

The joint MI between each detector configuration and the two target variables is summarized in Table 2. For ${\mathrm{StO}}_{2}$ prediction, the surface configuration alone yielded 0.1904 bits. The coaxial detector in isolation yielded 0.0000 bits with respect to ${\mathrm{StO}}_{2}$, indicating that the detected coaxial signal showed negligible marginal information about deep tissue oxygenation under the modeled geometry. This is consistent with the physical design intent: the detected coaxial signal is dominated by superficial photon paths and has limited sensitivity to hemoglobin absorption in the dermal capillary bed.

**Table 2:** Joint mutual information in bits for each detector configuration and target variable, estimated using the KSG estimator with k=5. The displayed conditional melanin gain is calculated from the displayed joint values.

Configuration	MI with ${\mathrm{StO}}_{2}$ (bits)	MI with melanin (bits)
Surface detectors only	0.1904	1.6149
Coaxial detector only	0.0000	1.8899
Surface + Coaxial detectors	0.2375	1.9421
Conditional gain of coaxial given surface detectors to detect ${\mathrm{StO}}_{2}$	0.0471	NA
Conditional gain of coaxial detector given surface detectors for melanin estimation	NA	0.3272

The combined surface-coaxial configuration yielded 0.2375 bits for ${\mathrm{StO}}_{2}$, representing a conditional gain of 0.0471 bits beyond the surface array alone. This result is not contradictory with the coaxial channel’s zero marginal MI for ${\mathrm{StO}}_{2}$. The coaxial channel does not encode oxygenation information directly; rather, it encodes epidermal melanin information that the surface detectors cannot fully resolve on their own. By providing the model with a cleaner characterization of the melanin confound, the coaxial channel indirectly reduces ambiguity in the surface signals, allowing more of its 0.1904 bits of ${\mathrm{StO}}_{2}$ information to be correctly interpreted. The conditional gain, therefore, reflects a reduction in interference rather than a direct addition of oxygenation sensitivity.

For melanin prediction, the coaxial detector alone yielded 1.8899 bits, exceeding the 1.6149 bits achieved by the complete surface detector array. The combined configuration yielded 1.9421 bits. The conditional gain calculated from these displayed values is 0.3272 bits, as shown in Eq. (13):

$$I(X_{C};M|X_{S})=1.9421-1.6149=0.3272\text{ bits}.$$ (13)

The relatively high mutual information obtained from the surface detector array may partly reflect the contribution of the shortest source-to-detector separation of 6.93 mm, which is expected to retain greater sensitivity to superficial epidermal absorption than the larger separations of 18.86 and 19.63 mm. However, because mutual information was estimated jointly using all three surface detector channels, the present analysis cannot quantitatively attribute the 1.6149 bits to the shortest separation alone. The asymmetry between the negligible marginal information of the coaxial detector for ${\mathrm{StO}}_{2}$ and its 1.8899 bits of information for melanin provides model independent evidence that the two detector geometries encode distinct and complementary aspects of the tissue optical state. The results are summarized in Table 2.

### 3.2 Hyperparameter Optimization

The randomized search over 540 configurations identified the following optimal parameter set: number of estimators = 1000, learning rate = 0.05, maximum tree depth = 3, row subsampling = 0.70, column subsampling = 0.80, L1 regularization = 0.10, L2 regularization = 1.0, and gamma = 0.

### 3.3 Model Performance

The trained XGBoost model was evaluated under LOGO cross-validation for each of the three detector configurations. The results are summarized in Table 3.

**Table 3:** Model performance for ${\mathrm{StO}}_{2}$ prediction under validation grouped by melanin volume fraction. Mean MSE, mean RMSE, and sample SD of fold RMSE are calculated from the nine displayed fold RMSE values in Table 4; $R^{2}$ values are retained from the reported model evaluation. Here, pp denotes percentage points on the 0 to 100 ${\mathrm{StO}}_{2}$ scale. MSE is expressed in squared percentage points, and $R^{2}$ is dimensionless.

Configuration	Average MSE (${\mathrm{pp}}^{2}$)	Average RMSE (pp)	$R^{2}$	RMSE SD (pp)
Surface detectors only	129.34	11.34	0.613	0.93
Coaxial detector only	484.83	21.65	-0.447	4.24
Surface + coaxial detectors	32.98	5.41	0.90	2.05

The hybrid surface plus coaxial configuration reduced mean RMSE by 52.3% relative to the surface only configuration, from 11.34 to 5.41 percentage points of ${\mathrm{StO}}_{2}$. The mean MSE, mean RMSE, and sample SD of fold RMSE in Table 3 were recalculated from the nine displayed fold RMSE values in Table 4 to ensure consistent aggregation.

**Table 4:** RMSE for each detector configuration at every melanin concentration held out during validation. The abbreviation pp denotes percentage points of ${\mathrm{StO}}_{2}$; melanin concentration is a volume percentage.

Held out melanin concentration (%)	Surface only RMSE (pp)	Coaxial only RMSE (pp)	Surface plus coaxial RMSE (pp)
5	11.57	31.42	9.00
10	11.05	18.82	8.06
15	11.36	18.60	3.76
20	11.59	20.46	4.00
25	11.52	25.61	5.60
30	11.62	19.13	3.68
35	12.34	19.44	3.50
40	11.95	21.32	4.44
45	9.05	20.08	6.62

The configuration using only the coaxial detector, with an RMSE of 21.65 percentage points of ${\mathrm{StO}}_{2}$, performed substantially worse than either configuration incorporating surface detectors. This supports the role of the coaxial channel as a complementary epidermal reference under the modeled geometry. Together, these results suggest that the coaxial channel is most useful when interpreted with spatially separated surface detectors, which contribute additional path length and depth sensitivity.

Figure 10 further supports this trend by showing that the combined surface and coaxial detector configuration produces predictions that cluster closely around the identity line across held-out melanin groups, indicating improved generalization and reduced pigmentation-dependent prediction bias. The figure presents the results obtained from the combined simulated surface and coaxial detector measurements under leave-one-group-out (LOGO) cross-validation experiments. Each color corresponds to an entire held-out melanin group containing the full range of simulated ${\mathrm{StO}}_{2}$ values, with the model trained exclusively on the remaining melanin groups and evaluated on previously unseen pigmentation levels.

**Fig. 10:**
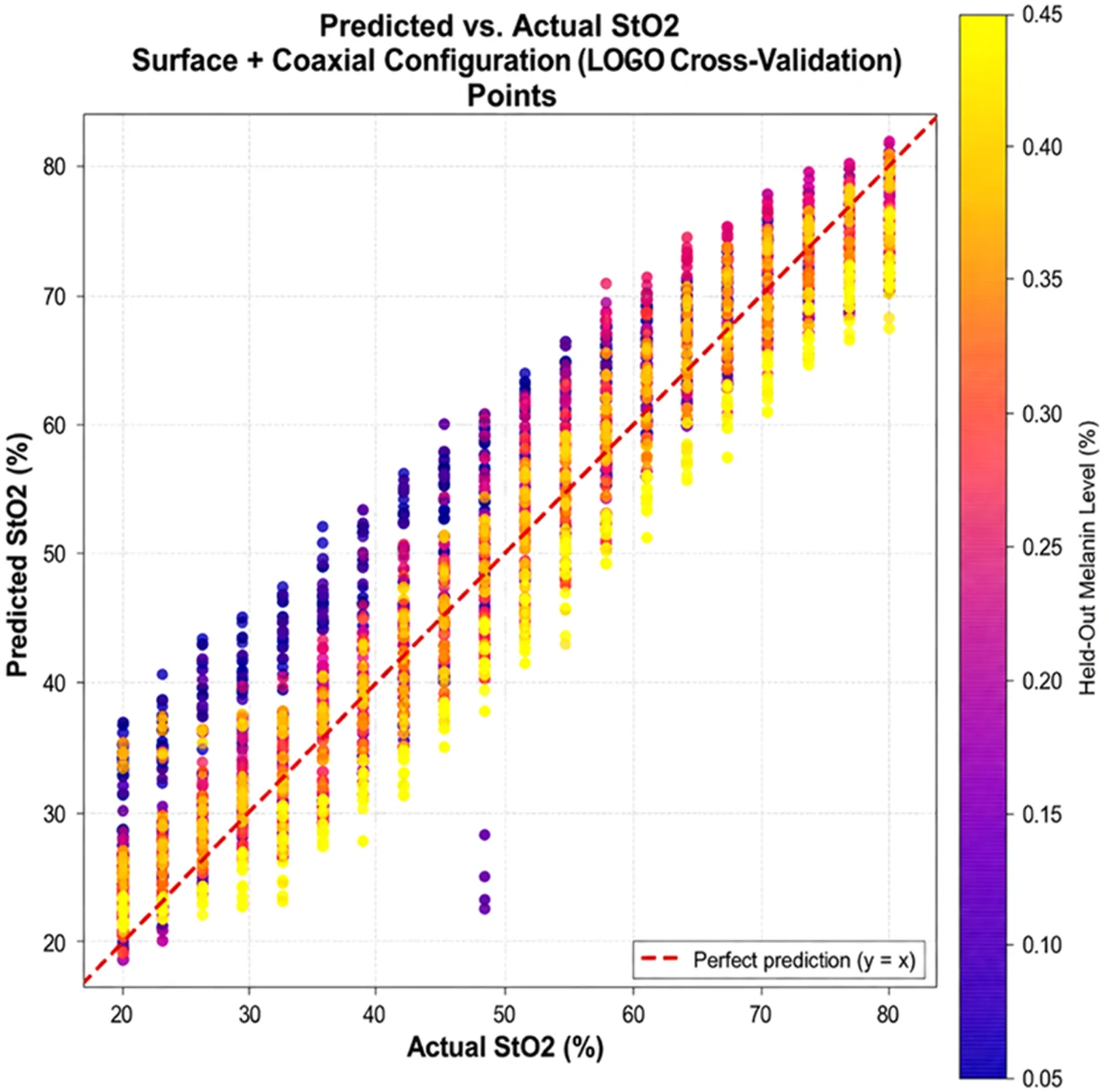
Predicted versus actual ${\mathrm{StO}}_{2}$ for the combined simulated surface and coaxial detector configuration under LOGO cross-validation. Each point represents a single out-of-fold prediction for a particular simulated ${\mathrm{StO}}_{2}$ value. The color corresponds to the melanin volume fraction of the held-out test group, where each test group contains the full range of simulated ${\mathrm{StO}}_{2}$ values.

The fold results indicate that the improvement was not driven by a single melanin group. Hybrid RMSE ranged from 3.50 to 9.00 percentage points of ${\mathrm{StO}}_{2}$, compared with 9.05 to 12.34 for the surface-only model and 18.60 to 31.42 for the coaxial-only model. Hybrid RMSE was 9.00 at 5% melanin and 8.06 at 10% melanin; it remained below 6.7 percentage points at the other seven melanin levels. The results are summarized in Table 4.

### 3.4 Exploratory Experimental Analysis of Coaxial Reflectance

The secondary experimental analysis compared absolute and baseline-referenced prediction from coaxial PhasorDetect measurements. Because each validation fold excluded one recording rather than an entire participant, the results characterize agreement for excluded recordings while the participant’s opposite-side recording remained available for training. They do not establish performance on entirely unseen participants or directly validate the proposed hybrid architecture.

The experimental LSBoost models used feature constructions related to those in the simulation study, with the distinctions described in Sec. 2.7. The results are summarized in Table 5 and Fig. 11. Absolute prediction yielded a pooled RMSE of 17.10 percentage points of ${\mathrm{StO}}_{2}$, an MAE of 12.56 percentage points, and an $R^{2}$ of −0.489. These values were verified from all 8148 measured and predicted pairs stored in the original MATLAB figure. The numerical errors from the simulation and experimental analyses are not directly comparable because the experimental data include variability in probe placement, contact pressure, tissue properties, device drift, synchronization, and measurement sites and use a different validation grouping.

**Table 5:** Experimental PhasorDetect performance under validation that excluded one recording at a time. The dataset contains 28 recordings from 14 participants; the opposite side recording from the same participant remained available for training. Metrics were verified from the original plotted predictions. Pooled RMSE and MAE are expressed in percentage points, abbreviated pp, and $R^{2}$ is dimensionless.

Feature set	Target	Pooled RMSE (pp)	Pooled MAE (pp)	$R^{2}$
Physically inspired absolute features	${\mathrm{StO}}_{2}$	17.10	12.56	−0.489
Baseline shifted physically inspired features	$\Delta {\mathrm{StO}}_{2}$	9.77	5.57	0.439

**Fig. 11:**
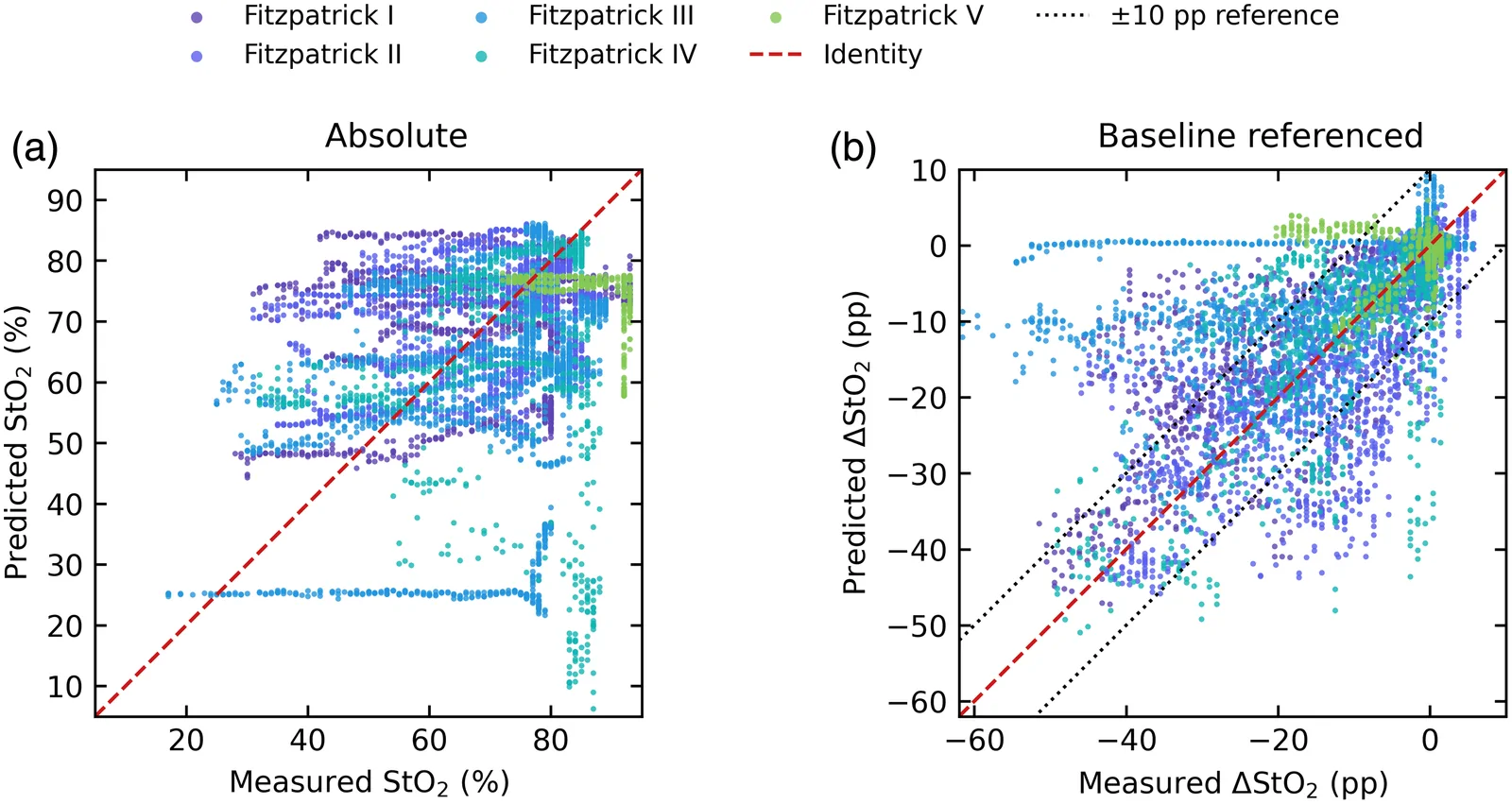
Measured and predicted oxygenation from the exploratory phasorDetect analysis. (a) Absolute ${\mathrm{StO}}_{2}$ prediction. (b) Baseline referenced $\Delta {\mathrm{StO}}_{2}$ prediction. Each panel contains the original 8148 plotted pairs from 28 recordings in 14 participants. Validation excluded one recording at a time; the same participant’s opposite side recording remained available for training. Colors indicate recorded Fitzpatrick type. The red dashed line is the identity line. Dotted boundaries in panel (b) indicate ±10 percentage points as an illustrative reference, not a confidence interval or a validated clinical acceptance threshold. The nearly horizontal type III series in panel (b) corresponds to the right-side recording of P009.

Baseline referencing improved pooled agreement for the excluded recordings: RMSE was 9.77 percentage points of $\Delta {\mathrm{StO}}_{2}$, MAE was 5.57 percentage points, and $R^{2}$ was 0.439. These values were verified from the original MATLAB figure coordinates. The improvement motivates further study of changes relative to baseline, but the residual dispersion and shared participant information across folds limit the interpretation. Independent participant validation is required before claiming generalization.

## 4 Discussion

The results show that adding a coaxial superficial reference channel to an optimized spatially resolved NIRS geometry substantially improves ${\mathrm{StO}}_{2}$ estimation across held-out melanin levels.

Because the illumination wavelengths, the tissue model, and the learning architecture were unchanged, the improvement is attributable to the added geometric information provided by the coaxial channel. This interpretation is also supported by the mutual information analysis.

### 4.1 Informational Role of the Coaxial Channel

The mutual information analysis clarifies the role of the coaxial channel. The coaxial detector carried negligible marginal information about ${\mathrm{StO}}_{2}$ but high information about melanin, whereas the surface detectors carried more direct oxygenation information. Thus, the coaxial channel improves ${\mathrm{StO}}_{2}$ prediction indirectly by helping resolve the superficial pigmentation component embedded in the spatially resolved signals.

The exploratory experimental analysis provides a limited in vivo counterpart to this interpretation. Absolute prediction from engineered coaxial reflectance features showed poor agreement, while baseline referencing improved pooled performance. These findings motivate further study of coaxial reflectance for changes relative to a baseline. However, validation excluded recordings rather than entire participants, so the results do not establish independent participant performance. They also do not directly validate the simulated hybrid geometry. The simulation and experimental comparisons therefore support further investigation of complementary optical measurements, with prospective participant validation needed to assess the proposed benefit in practice.

### 4.2 Physical Interpretation of the Coaxial Geometry

The informational asymmetry has a straightforward explanation rooted in photon transport theory. At the source-detector separations used by the surface detector array, detected photons follow curved paths that penetrate deeper into the dermis, with only a small portion of their path occurring within the epidermis. In contrast, the coaxial detector collects photons that have a short path length and are backscattered from superficial tissue layers, including the epidermal layer and uppermost dermis. As a result, a larger fraction of the detected photon path is spent within the epidermis, making the coaxial signal more sensitive to epidermal optical properties, including melanin.

The relationship between the detected signal and the underlying tissue optical properties can be described formally through the Jacobian sensitivity matrix J, with columns represent the sensitivity of each detector channel to perturbations in absorption and scattering at each tissue depth.^47^ In conventional spatially resolved configurations, the sensitivity vectors for absorption and reduced scattering at a given depth tend to be nearly collinear across detector channels, because all detectors sample overlapping photon path distributions through the same tissue volume. This Jacobian collinearity produces a state of parameter degeneracy in which the system cannot independently resolve whether an observed attenuation change originates from a change in epidermal melanin or a change in dermal hemoglobin oxygenation.

The coaxial detector may reduce this degeneracy by contributing a sensitivity profile that is less collinear with those of the distal channels. In practical terms, this additional superficial reference is expected to improve the conditioning of the inverse problem by reducing ambiguity between epidermal attenuation and deeper hemoglobin absorption. Because the mean photon path length for coaxial photons is expected to be shorter than that of the distal channels, the detected signal is more strongly weighted toward backscattering and superficial absorption than toward hemoglobin absorption in the deeper dermal vasculature. This coaxial measurement, therefore, functions as a baseline anchor; by providing a direct estimate of the superficial attenuation contribution at the point where pathlength is minimized, the system can reduce the ambiguity between epidermal confounding and deeper hemoglobin absorption in the distal signals. In inverse problem terms, the additional superficial reference is expected to improve the effective conditioning of the estimation problem, although explicit Jacobian conditioning analysis was not performed in this study.

This regime-level distinction is also consistent with sub-diffuse reflectance theory. At small source-detector separations, detected photons undergo relatively few scattering events and retain greater sensitivity to superficial absorption and scattering phase function effects than photons collected at larger separations. Thus, the coaxial channel is expected to encode epidermal attenuation and near-surface scattering information that is not redundant with the distal SRS channels.^47^

This distinction also clarifies the role of baseline correction. Baseline referencing can subtract fixed offsets produced by patient-specific pigmentation, coupling, and local tissue structure. However, it cannot create missing depth sensitivity. Once the measurement is expressed relative to baseline, the model can estimate changes in oxygenation more reliably, but the absolute baseline itself remains unknown. The proposed hybrid coaxial plus SRS geometry addresses this gap by combining two complementary sources of information: the coaxial channel provides superficial reference information, while the SRS channels provide deeper hemoglobin-sensitive measurements.

### 4.3 Experimental Motivation for Combining Coaxial and SRS Measurement.

In this exploratory analysis, referencing the engineered spectral features to baseline reduced pooled RMSE to 9.77 percentage points of $\Delta {\mathrm{StO}}_{2}$ and produced a positive $R^{2}$ under recording-based validation. This suggests that baseline referencing can reduce some fixed offsets within the present dataset. However, it does not establish generalization to unseen participants, and prediction of a change cannot determine whether the initial absolute oxygenation state is normal or pathological.

The nearly horizontal subset of $\Delta {\mathrm{StO}}_{2}$ predictions in Fig. 11 was traced to the right-side recording of participant P009, with recorded Fitzpatrick type was III. Matching the measured sequences in the MATLAB figure to the synchronized source data identified this recording unambiguously. Its predicted versus measured slope was approximately 0.002, and its RMSE was 23.93 percentage points, despite a measured range of 55 percentage points. Other recordings from participants with type III showed appreciable tracking, so the pattern does not establish that the entire skin type is unsuitable. Possible contributors include limited oxygenation sensitivity, baseline or tissue coupling differences, timing or measurement site mismatch, and compression of the regression output. These explanations are not distinguished by the present analysis. The observation therefore motivates examination of signal quality and validation in entirely excluded participants rather than a biological exclusion based on skin type.

This is the central motivation for the hybrid design evaluated in simulation. A coaxial channel alone is useful as a superficial reference and baseline anchor. An SRS channel alone remains vulnerable to shared superficial attenuation. The combined geometry provides both superficial reference information and deeper oxygenation-sensitive information, enabling the model to separate epidermal confounding from dermal hemoglobin absorption more effectively than either measurement class alone.

### 4.4 Clinical Implications for Equitable Tissue Oxygenation Measurement.

The clinical motivation for this work is the documented sensitivity of optical oximetry to skin pigmentation. Pulse oximetry has been shown to overestimate arterial oxygen saturation in some patients with darker skin tones,^18^ and error associated with pigmentation has also been reported for commercial NIRS oximeters.,^15^^–^^17^,^19^ The proposed geometry provides a channel that preferentially samples superficial optical properties, offering a reference for the pigmentation contribution in the deeper measurements. In Table 4, the hybrid configuration has a lower RMSE than either individual configuration at every tested melanin level. Its RMSE is below 6.7 percentage points at seven of the nine levels, with larger residual errors at 5% and 10% melanin. These simulation findings motivate broader experimental evaluation; they do not establish uniform clinical accuracy across skin types.

### 4.5 Limitations of the Study.

Several limitations should be acknowledged. First, the tissue model is a deliberate simplification that excludes hair follicles, sweat glands, and heterogeneous dermal vasculature. The capillary plexus was modeled as a homogeneous layer with a uniform blood volume fraction rather than a discrete vessel network. Although common in prior literature, these simplifications isolate the effect of probe geometry on melanin generalization but may not capture all sources of signal variance present *in vivo*.^48^

Second, the experimental analysis was exploratory and did not validate the full coaxial plus SRS architecture. The experimental device lacked the simulated SRS detector array. In addition, validation grouped observations by recording rather than by participant: the opposite-side recording from the same participant could enter the training set. This dependence can make performance more favorable than evaluation on entirely unseen participants. The reported experimental errors should therefore be interpreted as descriptive results under recording-based validation. Validation that excludes both sides of each participant together, followed by an independent cohort, is needed to assess generalization.

Third, the experimental shifted models estimate $\Delta {\mathrm{StO}}_{2}$ relative to a reference baseline rather than absolute ${\mathrm{StO}}_{2}$. The first 100 retained observations were used to define that baseline and were also included in the evaluation. The analysis therefore evaluates retrospective agreement under a defined baseline procedure, not prospective clinical performance or an independently recovered absolute oxygenation state. Future studies should define baseline acquisition prospectively and assess its stability.

### 4.6 Future Work

Our group is currently developing a next-generation PhasorDetect probe that integrates both geometries evaluated here, combining the existing coaxial LED–photodetector channel with a spatially resolved detector array around the cone aperture. Planned validation will proceed from layered tissue-simulating phantoms with controlled melanin-equivalent absorbers to vascular occlusion testing across an expanded Fitzpatrick range, enabling direct experimental assessment of the melanin decoupling mechanism predicted by the simulations.

A further simulation study could generate independent training and test datasets that span the same melanin range and compare their performance with an evaluation in which an entire melanin level is held out. This comparison would help distinguish error associated with extrapolation to an unseen melanin level from error that persists when that level is represented during training. These additional experiments were suggested during review and remain future work.

Therefore, the present study establishes a mechanistic and computational basis for coaxial-assisted melanin correction, while future prototype studies are needed to determine whether the same improvement is retained under physical probe contact, anatomical variability, and real tissue heterogeneity.

## 5 Conclusion

This study evaluated whether adding a coaxial detector to a spatially resolved near-infrared spectroscopy probe improves the accuracy and melanin robustness of tissue oxygen saturation estimation in a simulated multilayer tissue model. Using a three-dimensional Monte Carlo simulation framework spanning a physiologically representative range of epidermal melanin concentrations, epidermal thicknesses, and ${\mathrm{StO}}_{2}$ levels, a surface-only SRS configuration was compared against a hybrid coaxial plus SRS configuration.

The hybrid configuration exhibits a 52.3% reduction in prediction errors compared to the SRS-alone detector array. Mutual information analysis confirmed that this improvement is mechanistically grounded: the coaxial detector carries 1.8899 bits of information about epidermal melanin, exceeding the full surface array, while carrying negligible direct information about ${\mathrm{StO}}_{2}$. This asymmetry identifies the coaxial channel as a geometrically selective epidermal reference sensor with primary function is to resolve the melanin component of the surface signals, enabling the predictive model to decouple epidermal absorption from the deeper hemoglobin oxygenation signal.

The exploratory PhasorDetect analysis showed poor absolute agreement, with pooled RMSE near 17 percentage points and negative $R^{2}$. Baseline referenced features reduced pooled RMSE to ∼10 percentage points and produced positive $R^{2}$, although a recording showed nearly constant predictions. These results were obtained with recording-based validation, in which the opposite side from the same participant remained available for training. They motivate further work but do not demonstrate performance on unseen participants. Estimation of $\Delta {\mathrm{StO}}_{2}$ also does not resolve absolute quantification.

Therefore, the proposed coaxial plus SRS architecture is motivated by both simulation and experimental evidence: coaxial measurements provide superficial reference information and support baseline relative monitoring, but robust absolute ${\mathrm{StO}}_{2}$ estimation requires combining that reference with depth-sensitive spatially resolved measurements.

## Data Availability

Simulation data and code resources are provided in the project repository: https://github.com/vedanshipatel30/JBO-260172. Requests for the experimental recordings, associated participant metadata, and additional analysis materials should be directed to the corresponding author and are subject to applicable consent and institutional requirements.
